# Effect of Strain Rates on the Stress–Strain Behavior of FRP-Confined Pre-Damaged Concrete

**DOI:** 10.3390/ma13051078

**Published:** 2020-02-28

**Authors:** Yugui Cao, Muyu Liu, Yang Zhang, Jun Hu, Shengchun Yang

**Affiliations:** 1 Hubei Key Laboratory of Roadway Bridge and Structure Engineering, Wuhan University of Technology, Wuhan 430070, China; liumuyu@whut.edu.cn (M.L.); zhangyang280279@whut.edu.cn (Y.Z.); 2 College of Water and Architectural Engineering, Shihezi University, Shihezi 832003, China; hujun@hust.edu.cn; 3 College of Civil Engineering, Inner Mongolia University of Science and Technology, Baotou 014010, China; ysc03045@126.com

**Keywords:** pre-damaged concrete, confinement, strain rate, stress–strain behavior

## Abstract

There are many studies on fiber-reinforced polymer (FRP)-confined pre-damaged concrete under quasi-static strain rates. However, few studies have focused on FRP-confined pre-damaged concrete under high strain rates. Thus, an experimental and analytical investigation was conducted to obtain the mechanical behavior of FRP-confined pre-damaged concrete under different strain rates. The results show that the stress–strain curves, ultimate stress, and strain values were affected by strain rate and the extent of concrete damage. A stress–strain model of FRP-confined pre-damaged concrete considering the strain rate was developed by modifying a stress–strain model of FRP-confined pre-damaged concrete under quasi-static loading. The proposed model was evaluated by using test data. The evaluation results show that the proposed model can predict the stress–strain behavior of FRP-confined pre-damaged concrete under different strain rates.

## 1. Introduction

At present, a large number of concrete structures need to be strengthened due to aging, improper maintenance, or earthquake damage. As a kind of reinforcement material, fiber-reinforced polymer (FRP) is widely used in repairing and strengthening damaged concrete columns [1,2,3,4,5,6,7], beams, girders, and slabs [8], owing to its advantages of light weight, corrosion resistance, and convenient construction. Extensive studies have been conducted on the mechanical behavior of FRP-confined intact concrete under quasi-static loading, and more than 90 stress–strain models have been proposed [9,10].

The existing literature concludes that the mechanical behaviors of FRP-confined intact concrete and damaged concrete are different [11,12,13,14,15,16]. As the degree of concrete damage increases, the compressive strength of FRP-confined pre-damaged concrete decreases. Some studies [11,12,15,16,17,18] indicated that FRP could significantly improve the bearing and deformational capacity of pre-damaged concrete cylinders, and that the degree of concrete damage had a significant effect on the stress–strain curves of the FRP-confined concrete. Because the existing stress–strain models could not provide sufficiently accurate calculation results, Wu et al. [11] further studied the mechanical behavior of FRP-confined pre-damaged circular concrete and proposed a stress–strain model of FRP-confined pre-damaged concrete. Subsequently, Li et al. [12] proposed a stress–strain model of FRP-confined pre-damaged concrete with a square cross-section by modifying Wu et al.’s model [11]. However, Wu et al. and Li et al.’s models are only suitable for FRP-confined pre-damaged concrete columns under quasi-static strain rates, and there are no related tests on FRP-confined pre-damaged concrete columns under high strain rates [11,12].

It is well known that concrete is a strain rate-sensitive material. As the strain rate increases, the compressive strength of the concrete increases. This phenomenon is caused by the contact surface restriction and the lateral inertia of materials during dynamic compression [19,20]. For FRP-confined concrete columns, FRP can also provide lateral confinement on the lateral surface of the concrete. Therefore, FRP-confined concrete is also a strain rate-sensitive material. Some experimental tests have been conducted on FRP-confined intact concrete under different strain rates [21,22,23,24,25,26]. These test results showed that the strain rates had a significant effect on the stress–strain relationship, compressive strength, and ultimate strain of the FRP-confined concrete, and stress–strain models for FRP-confined intact concrete columns were proposed [23,25,27]. However, to the best of our knowledge, there are no related studies on FRP-confined pre-damaged concrete columns under different strain rates.

To address the research gap of FRP-confined pre-damaged concrete under different strain rates, a comprehensive test for FRP-confined pre-damaged concrete with varying levels of damage and strain rates was conducted in this paper. The effect of the strain rate combined with the concrete damage level on the stress–strain relationship of FRP-confined concrete was experimentally studied. Finally, a stress–strain model was developed by modifying Wu et al.’s model [11]. The evaluation results show that the proposed model can be used to predict the stress–strain behavior of FRP-confined pre-damaged concrete under different strain rates.

## 2. Strain Rate Effect and Definition of Concrete Damage

**Figure 1:**
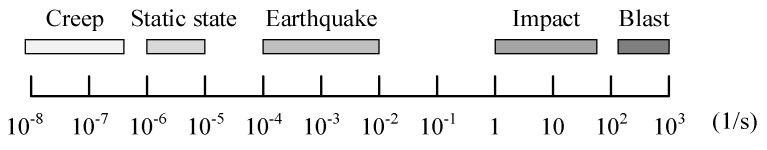
The strain rates in different kinds of loads.

During an earthquake, concrete structures will withstand larger strain rates than static values. The relationship between strain rates and load conditions, which is defined by Bischoff and Perry [28], is shown in Figure 1.

**Figure 2:**
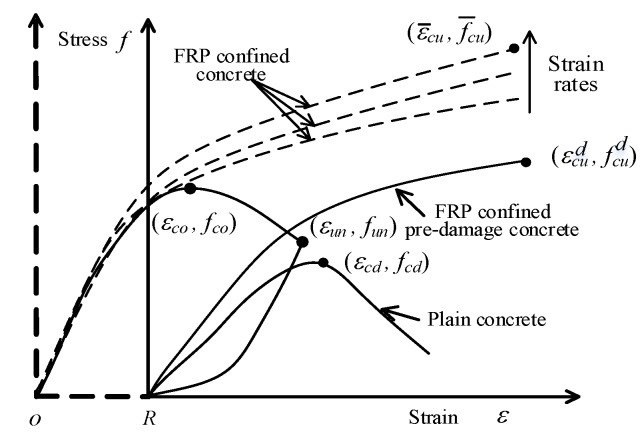
Typical stress–strain curves.

It is known that the strain rate has a great effect on the stress–strain curve of FRP-confined concrete [21,23,26]. With increasing strain rate, the strain hardening stiffness and compressive strength also increase. In Figure 2, the dashed lines show the stress–strain relationship of FRP-confined concrete under different strain rates.

Concrete damage also has an effect on the compressive strength, ultimate strain, and stress–strain relationship of FRP-confined concrete [11,12,15]. The typical stress–strain curve of FRP-confined pre-damaged concrete is shown in Figure 2. Wu et al. [11] defined the concrete damage degree *δ* by using Equation (1):

(1) $$\delta =1-\frac{f_{cd}}{f_{co}}$$

where *f_cd_* and *f_co_* are the reloading stress value and compressive strength of plain concrete, respectively, as shown in Figure 2.

For the FRP-confined pre-damaged concrete, only the unloading stress *f_un_* value of plain concrete was measured. The reloading peak stress of plain concrete *f_cd_* could not be directly obtained from the test. Therefore, Wu et al. [11] developed a relationship between the unloading stress value *f_un_* and the reloading peak stress value *f_cd_*, as shown in Equations (2a) and (2b):

(2a) $$\frac{f_{cd}}{f_{co}}=1-0.029\frac{f_{un}}{f_{co}},\;\mathrm{when}\;\varepsilon_{\mathrm{un}}\;\leq \;\varepsilon_{\mathrm{co}}$$

(2b) $$\frac{f_{cd}}{f_{co}}=0.64\left(1+\frac{f_{co}-f_{un}}{f_{co}}\right)-2.72\left(1+\frac{f_{co}-f_{un}}{f_{co}}\right)+3.07,\;\mathrm{when}\;\varepsilon_{\mathrm{un}}\;>\;\varepsilon_{\mathrm{co}}$$

where *ε_un_* and *ε_co_* are the unloading strain value and peak strain value of the plain concrete, respectively, as shown in Figure 2. Recently, Li et al. [12] verified the accuracy of Equations (2a) and (2b) by using their own experimental data. Therefore, Equations (2a) and (2b) are used to calculate the reloading peak strength *f_cd_* in this work.

## 3. Experimental Program

### 3.1. Specimen Design

**Figure 3:**
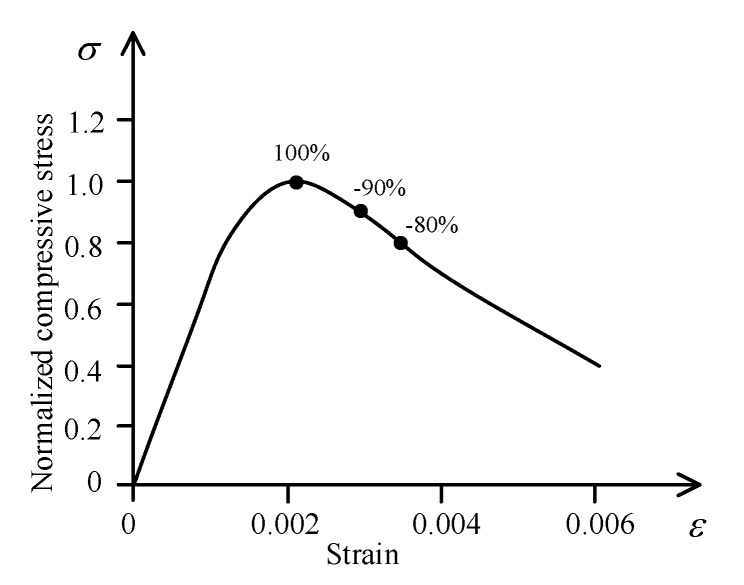
Definition of pre-damage levels (λ).

A total of 28 circular cylinders, 150 mm in height and 300 mm in diameter, were cast at one time. Two identical specimens were cast for each design. The specimens were divided into three groups based on the pre-damage levels (100%, −90%, and −80%). The definition of the concrete pre-damage level (λ) is the ratio of the unloading stress (*f_un_*) to the concrete compressive strength *f_co_*, where the negative sign means unloading stress on the descending branch; the three pre-damage levels are shown in Figure 3. For example, a pre-damage level of 100% means the pre-damage load is 100% of unconfined concrete strength before wrapping. The pre-damaged concrete was wrapped with 1 and 2 layers of FRP jackets. It can be seen from Figure 1 that strain rates of 3.33 × 10^−5^/s, 3.33 × 10^−4^/s, and 3.33 × 10^−3^/s are related to load conditions ranging from static to earthquake. In this test, these strain rates were also used to study the mechanical behavior of the FRP-confined pre-damaged concrete under earthquake loadings.

The detailed parameters of all the specimens are listed in Table 1. In Table 1, the specimen ID is composed of letters and numbers. The first letter and number denote the concrete pre-damage level; D0, D1, D2, and D3 denote pre-damage levels of 0%, 100%, −90%, and −80%, respectively. The second letter and number (L1 or L2) denote the number of FRP layers. The third letter and number denote the strain rate; S1, S2, and S3 denote the strain rates of 3.33 × 10^−5^/s, 3.33 × 10^−4^/s, and 3.33 × 10^−3^/s, respectively. The last number denotes the specimen batch. For example, D1L2S2-1 indicates the specimen in batch 1 with a concrete pre-damage level of 100%, 2-ply FRP, and a strain rate of 3.33 × 10^−4^/s. In Table 1, *f_cu_* and *ε_cu_* are the ultimate stress and strain, respectively; the definitions of *E*_1_, *n*, *f_o_*, and *E*_2_ are given in Section 5.

### 3.2. Material Properties

To reduce the compressive strength dispersion of the plain concrete cylinders, all the specimens were cast at one time with commercial concrete. The water/cement ratio was 0.47, and the compressive strength of the cylinder was 27 MPa.

A two-part epoxy resin was used as the adhesive. Carbon fiber-reinforced polymer (CFRP) with a thickness of 0.167 mm was used to repair the damaged concrete. The CFRP material properties were obtained by using the flat coupon test under three strain rates. Material properties of CFRP are listed in Table 2. It can be seen that strain rate had an insignificant effect on the mechanical properties of CFRP. Therefore, the average values in Table 2 were used in the following section. 

### 3.3. Specimen Preparation

The concrete cylinders were cast at the structural engineering laboratory of Wuhan University of Technology. The specimens were prepared using the following steps: (1) The pre-damage process was carried out by using a 2000 kN compression machine under a displacement rate of 3.3 × 10^−5^/s. (2) The surface of the specimen was carefully cleaned using sandpaper. (3) A single or double layer of the CFRP jacket was wrapped around the specimen, and each layer had a single overlap of 150 mm in length. It should be noted that the concrete cracks were not repaired by glue injection. To avoid premature failure, additional FRP sheets with a 30 mm width were used to strengthen both ends of the specimen. (4) One layer of plastic film was used to wrap the specimen to guarantee a tight wrap. The plastic film was removed before the test.

### 3.4. Testing Setup and Testing Program

**Figure 4:**
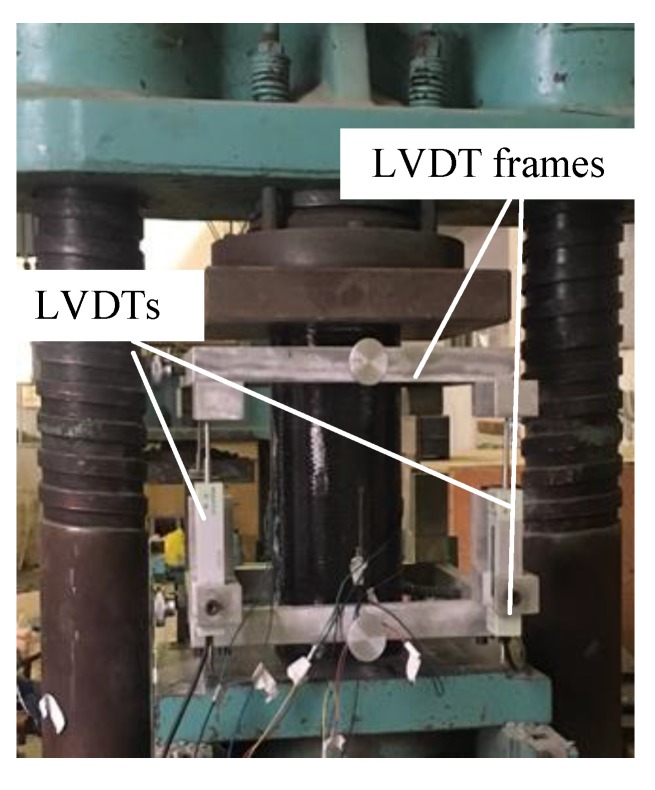
Test setup.

Four travel linear variable differential transformers (LVDTs) and two strain gauges were used to measure the axial displacement and axial strain of the specimens, respectively. The strain gauges and LVDTs were all installed at the middle height of the specimen. All test data were recorded by an automatic data acquisition system. Axial compression on the specimens was applied by a compression machine, as shown in Figure 4. All the specimens were tested under monotonic loading until failure with strain rates of 3.33 × 10^−3^/s, 3.33 × 10^−4^/s, and 3.33 × 10^−5^/s.

## 4. Test Results and Analysis

### 4.1. Failure Modes

**Figure 5:**
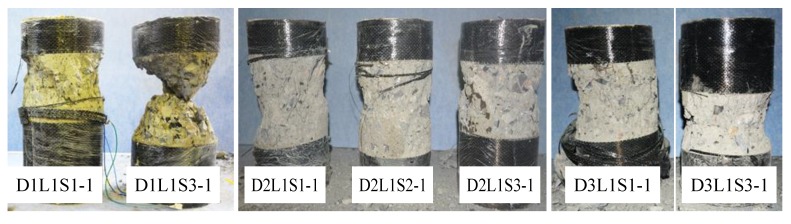
Typical failure of specimens.

When the applied load was close to the carrying capacity of specimen, clicking sounds could be heard, and the final failure occurred suddenly with an explosive sound accompanied by flying fragments of concrete and CFRP, regardless of the damage level or strain rate. As the loading rate increased, the loudness of the explosion of the specimen gradually increased, and the explosion sound of the two-layer FRP-confined concrete was louder than that of the one-layer CFRP. The explosion sound for the specimen under a strain rate of 3.33 × 10^−3^/s was the loudest, while the explosion sound for the specimen under a strain rate of 3.33 × 10^−5^/s was the softest. All the failure states of the specimens occurred with the rupturing of the FRP sheets at the middle height of the specimens. Figure 5 shows the typical failure of specimens. It can be seen that the pre-damage level and strain rate had an insignificant effect on the damaged zones of the specimens.

### 4.2. Stress–Strain Responses

**Figure 6:**
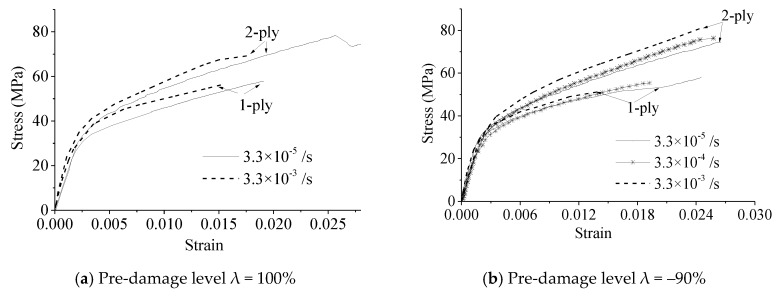

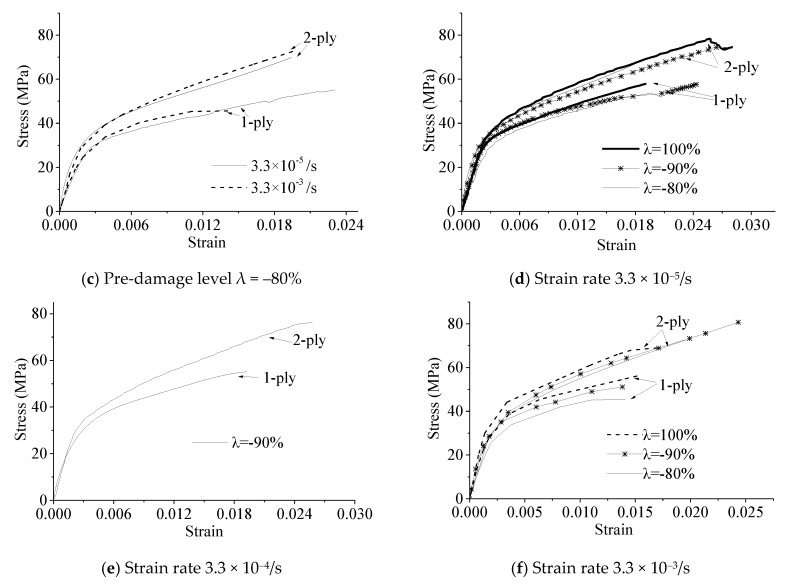
Effect of strain rates on the stress–strain curves.

The experimental stress–strain curves of the FRP-confined pre-damaged concrete are shown in Figure 6. The strain values were obtained by dividing the LVDT values by the center distances of the LVDT frames, while the stress values were obtained by dividing the compression machine readings by the cross-sectional areas of the specimens.

The experimental stress–strain curves of the specimens under different strain rates but the same damage levels are shown in Figure 6a–c. It can be seen that the strain rates had significant effects on the initial Young’s moduli and the shapes of the entire stress–strain curves. With increasing strain rate, the initial elastic stiffness increased. This phenomenon is consistent with the conclusions reported in [26,30]. Therefore, the strain rate had a significant effect on the stress–strain relationship of the FRP-confined damaged concrete and undamaged concrete columns.

The experimental stress–strain curves of the FRP-confined pre-damaged concrete with varying levels of pre-damage under the same strain rate are shown in Figure 6d–f. It can be seen that the pre-damage level *λ* had effects on the initial Young’s moduli and hardening stiffness of the stress–strain curves. As the concrete damage level *λ* increased, the initial stiffness and hardening stiffness of the stress–strain curves decreased. This phenomenon is consistent with the conclusion reported in other papers [11,12,13]. In other words, the concrete damage level had an effect on the stress–strain relationship in FRP-constrained concrete columns under different strain rates.

### 4.3. The Ultimate Condition

**Figure 7:**
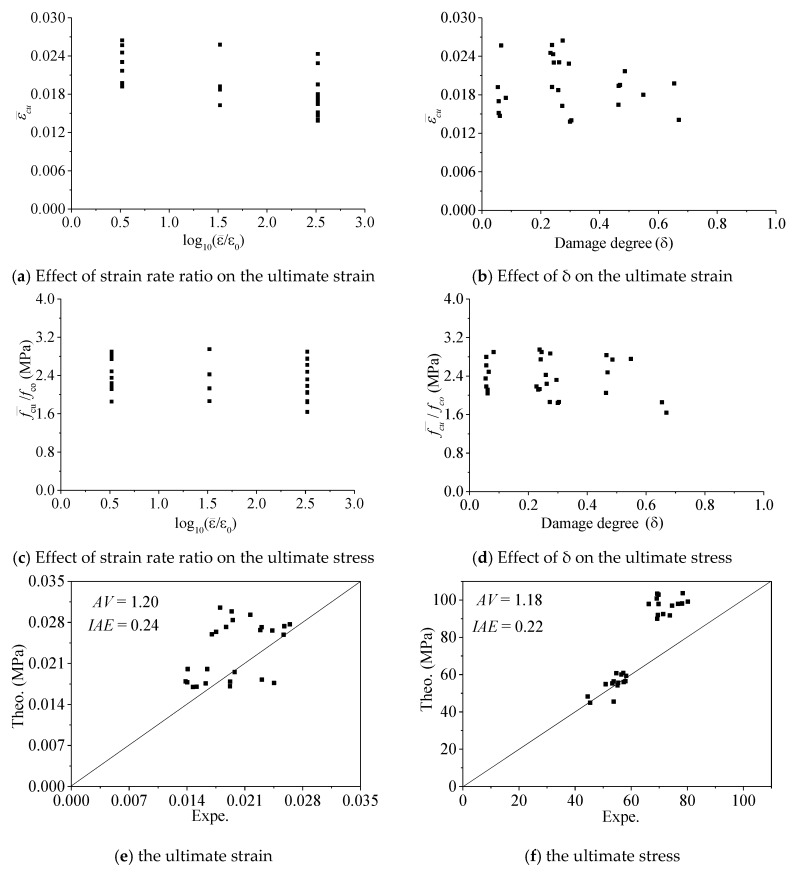
The ultimate state.

The ultimate strain and stress of FRP-confined pre-damaged concrete is shown in Figure 7. It can be seen from Figure 7a that the strain rate had a significant effect on the ultimate strain; its value decreased as the strain rate increased. This conclusion is consistent with results of FRP-confined intact concrete under different strain rates [11,21,22]. However, Figure 7c shows that strain rate ratio had an insignificant effect on ultimate stress. Figure 7b,d shows that concrete damage degree *δ* also had an effect on the ultimate strain and stress. Ultimate strain and stress values decreased with increases in the damage degree *δ*; Wu et al. also obtained the same conclusion [11].

Demir et al. [21,22] experimentally investigated only the mechanical behavior of FRP-confined concrete under different strain rates. Wu et al. [11] analyzed the mechanical behavior of FRP-confined concrete only under quasi-static loading and proposed an ultimate stress and strain model. There is not an existing ultimate strain and stress model for the FRP-confined pre-damaged concrete under different strain rates. Therefore, the test results were only compared with the calculated results of Wu et al.’s model [11]. The compared results are shown in Figure 7e,f, in which the error index *AV* values were 0.24 and 0.22 for ultimate strain and stress, respectively, and IAE values were 0.24 and 0.22 for ultimate strain and stress, respectively. The error indexes *AV* and *IAE* [2,9,31,32,33] are defined in Equations (3) and (4), respectively. It can be seen that there is an error between the theoretical value and the experimental value. The reason for this may be that the Wu et al. model [11] does not consider the strain rate.

(3) AV=∑(Theo.Expe.)n

(4) $$IAE=\frac{\sum \left|Theo.-Expe.\right|}{\sum \left|Expe.\right|}$$

where *Theo*. is the value calculated by the model, *Expe*. is the experimental value, and *n* is the test data number.

## 5. Proposed Stress–Strain Model

**Figure 8:**
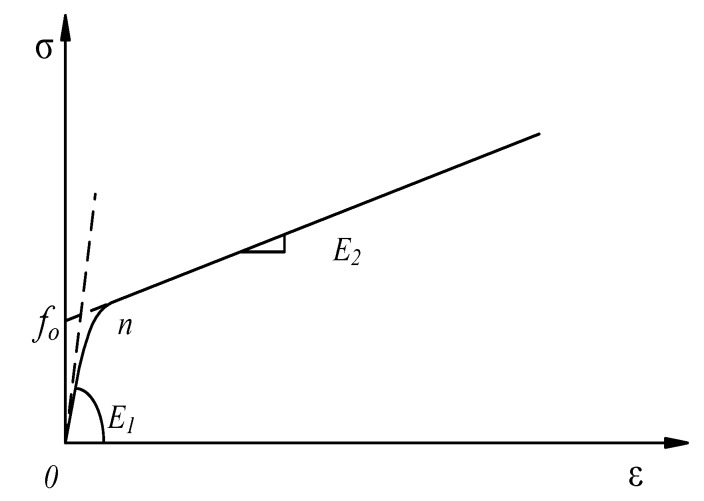
Typical stress–strain relationship.

Zhou and Wu [34] developed a continuous mathematical expression, given by Equation (5). This function can be used to describe the stress–strain relationship of FRP-confined intact concrete columns under different loading paths [10,35] and the stress–strain relationship of FRP-confined pre-damaged concrete columns under quasi-static strain rates [11,12]:

(5) $$f_{c}=\left(\left(E_{1}\varepsilon_{n}-f_{o}\right)e^{-\frac{\varepsilon_{c}}{\varepsilon_{n}}}+f_{o}+E_{2}\varepsilon_{c}\right)\left(1-e^{-\frac{\varepsilon_{c}}{\varepsilon_{n}}}\right)$$

where *E*_1_ and *E*_2_ are the initial stiffness and hardening stiffness for the stress–strain curve, respectively; *f_o_* is the intersection stress between the strain hardening curve and the vertical axis; and *ε*_n_ = *n* × *ε_o_*, *ε_o_* = *f_o_*/*E*_1_, and factor *n* control the curvature of the transfer part, satisfying 0 < *n* ≤ 1, as shown in Figure 8.

There are four parameters in Equation (5), namely *f_o_*, *E*_1_, n, and *E*_2_. These parameter values can be obtained by regressing the experimental stress–strain curves using Equation (4). For the intact concrete confined by FRP under quasi-static loading conditions, the values of the four parameters are determined by regression of the theoretical stress–strain curves, which are determined by using Hu and Wang’s model [29]. The values of the four parameters (*f_o_*, *E*_1_, *n*, *E*_2_) are listed in Table 1. For FRP-confined pre-damaged concrete, the four parameters (*f_o_*, *E*_1_, n, *E*_2_) become ${\bar{f}}_{o}$, ${\bar{E}}_{1}$, ${\bar{E}}_{2}$ and $\bar{n}$. The four parameters (${\bar{f}}_{o}$, ${\bar{E}}_{1}$, ${\bar{E}}_{2}$, $\bar{n}$) are also listed in Table 1. 

### 5.1. Analytical Stress–Strain Modeling

#### 5.1.1. Initial Elastic Modulus ${\bar{E}}_{1}$

**Figure 9:**
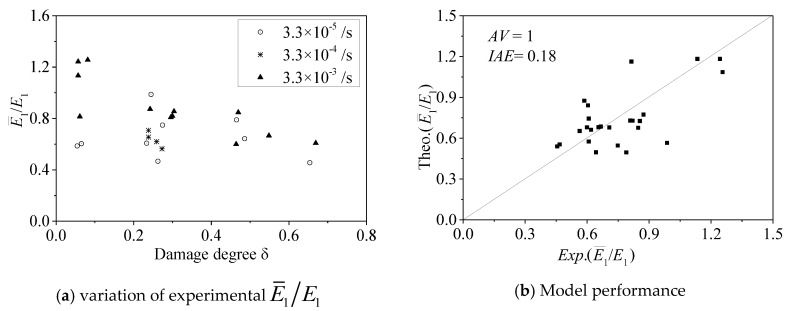
Initial slope ${\bar{E}}_{1}$.

Figure 9a shows the relationship between ${\bar{E}}_{1}/E_{1}$, the concrete damage degree *δ*, and the strain rate ratio $\dot{\varepsilon }/\varepsilon_{0}$, in which *E*_1_ is the initial elastic modulus of the FRP-confined intact concrete under quasi-static loading. Figure 9a shows that the concrete damage degree *δ* has a significant effect on the initial elastic modulus ratio ${\bar{E}}_{1}/E_{1}$. With increasing concrete damage degree *δ*, the initial elastic modulus ratio ${\bar{E}}_{1}/E_{1}$ value decreases. This phenomenon is consistent with the conclusion reported in [11,12]. Figure 9a also shows that the strain rate has a significant effect on the initial elastic modulus ratio ${\bar{E}}_{1}/E_{1}$. With increasing strain rate, the initial elastic modulus ratio ${\bar{E}}_{1}/E_{1}$ value increases. Other studies [21,26] also reached the same conclusion. Wu et al. [11] further proposed an initial stiffness model of FRP-confined pre-damaged concrete under static strain rate, as shown in Equation (6):

(6) $$\frac{{\bar{E}}_{1}}{E_{1}}=e^{\left(3.9\delta^{1.38}-1.65\delta \right){\left(\frac{f_{l}}{f_{co}}\right)}^{-0.106}-2.62\delta^{0.76}}$$

where *f_l_* is the FRP-confinement pressure. Recently, Li et al. [12] verified the accuracy of the initial stiffness model by using their own experimental data. Therefore, the initial elastic modulus model of FRP-confined pre-damaged concrete under different strain rates. ${\bar{E}}_{1}$ can be obtained in this work by modifying Wu et al.’s model [11] as follows:

(7) $$\frac{{\bar{E}}_{1}}{E_{1}}=e^{\left(3.9\delta^{1.38}-1.65\delta \right){\left(\frac{f_{l}}{f_{co}}\right)}^{-0.016}-2.62\delta^{0.76}+0.27{\left(\log_{10}\left(\frac{\dot{\varepsilon }}{\varepsilon_{0}}\right)\right)}^{0.65}}$$

When the strain rate $\dot{\varepsilon }$ is equal to *ε*_0_ (10^−5^/s), Equation (7) degenerates to Equation (6). Therefore, Equation (7) is reasonable.

The performance of Equation (7) is shown in Figure 9b, in which *AV* = 1 and *IAE* = 0.18. Equation (7) has good performance in predicting the initial elastic modulus value.

#### 5.1.2. Transition Curve Parameter $\bar{n}$

**Figure 10:**
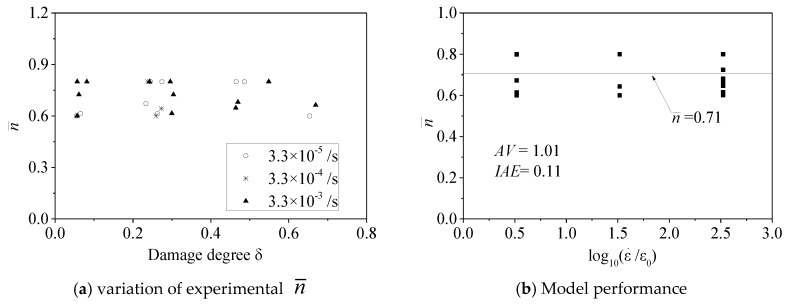
Evaluation of $\bar{n}$.

Figure 10a shows the relationship between the experimental value of parameter $\bar{n}$ and the strain rate ratio ${\bar{\varepsilon }}_{o}/\varepsilon_{o}$. It can be observed that the strain rate ratio and damage degree did not affect the parameter $\bar{n}$ value. Parameter $\bar{n}$ should be constant. Recent studies also indicated that the FRP-confinement stiffness, concrete compressive strength, and concrete damage level did not affect the parameter *n* value [11,12,34,36]. The performance of the $\bar{n}$ value is shown in Figure 10b, in which *AV* = 1.01 and *IAE* = 0.11. Therefore, a constant value of 0.71 was adopted for the parameter $\bar{n}$ value.

#### 5.1.3. Elastic Limit stress ${\bar{f}}_{o}$

**Figure 11:**
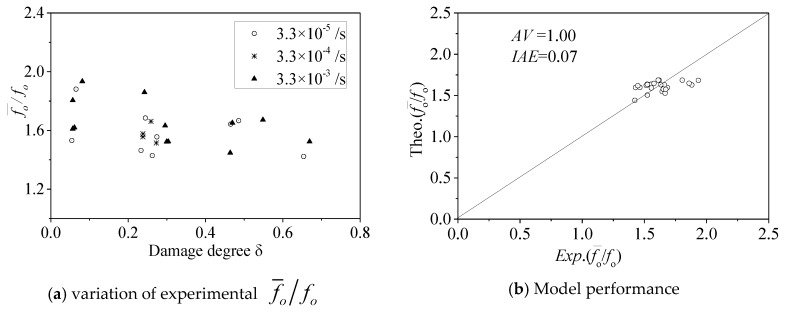
Elastic limit ${\bar{f}}_{o}$.

Figure 11a shows that the damage degree *δ* has an effect on the elastic limit ratio ${\bar{f}}_{o}/f_{o}$. With increasing concrete damage degree *δ*, the elastic limit ratio ${\bar{f}}_{o}/f_{o}$ decreases. Based on the experimental data of FRP-confined pre-damaged concrete under quasi-static loading, Wu et al. [11,12] also found that the damage degree had a significant effect on the parameter value of the elastic limit ratio ${\bar{f}}_{o}/f_{o}$, and the following function was developed:

(8) $$\frac{{\bar{f}}_{o}}{f_{o}}=1-0.3{\left(\frac{f_{co}}{f_{30}}\delta \right)}^{2}-0.12\left(\frac{f_{co}}{f_{30}}\delta \right)$$

where *f*_30_ is the concrete compressive strength of 30 MPa.

Figure 11a also shows that the strain rate ratio ${\bar{\varepsilon }}_{o}/\varepsilon_{o}$ has an effect on the elastic limit ratio ${\bar{f}}_{o}/f_{o}$. The elastic limit stress model ${\bar{f}}_{o}$ can be obtained by modifying Wu et al.’s model [11] as follows:

(9) $$\frac{{\bar{f}}_{o}}{f_{o}}=1-0.3{\left(\frac{f_{co}}{f_{30}}\delta \right)}^{2}-0.12\left(\frac{f_{co}}{f_{30}}\delta \right)+0.66{\left(\log_{10}\left(\frac{\dot{\varepsilon }}{\varepsilon_{0}}\right)\right)}^{0.05}$$

When the strain rate $\dot{\varepsilon }$ is equal to *ε*_0_ (10^−5^/s), Equation (9) degenerates to the elastic limit stress of the confined concrete under quasi-static loading. Therefore, Equation (9) is reasonable. The performance of Equation (9) is shown in Figure 11b, in which *AV* = 1.00 and *IAE* = 0.07. Equation (9) can be used to calculate the elastic limit stress value ${\bar{f}}_{o}$.

#### 5.1.4. Hardening Modulus ${\bar{E}}_{2}$

**Figure 12:**
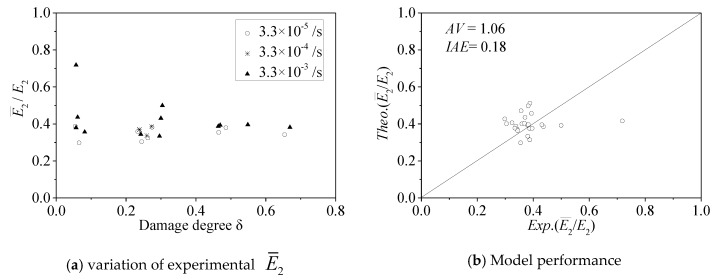
Hardening modulus ${\bar{E}}_{2}$.

The test results of the hardening modulus ratio ${\bar{E}}_{2}/E_{2}$ are shown in Figure 12a. It can be seen that the hardening modulus ratio ${\bar{E}}_{2}/E_{2}$ decreases with increasing damage degree *δ*. This conclusion is consistent with existing reports [11,12,13], which found that the damage degree *δ* and concrete strength ratio $f_{co}/f_{30}$ had a significant effect on the parameter value of the hardening modulus ratio ${\bar{E}}_{2}/E_{2}$, and a hardening modulus function was developed by Wu et al. [11] as follows:

(10) $$\frac{{\bar{E}}_{2}}{E_{2}}={\left(\frac{f_{co}}{f_{30}}\right)}^{2.76\delta }-0.46\delta$$

Figure 12a shows that the hardening modulus ratio ${\bar{E}}_{2}/E_{2}$ decreases with increasing strain rate ratio. Therefore, the effect of the strain rate should be considered in the model of the hardening modulus ${\bar{E}}_{2}$. The following equation can be obtained by modifying Wu et al.’s model [11]:

(11) E¯2E2=(fcof30(1+0.24(log10(ε˙ε0))0.37))2.67δ−0.46δ−0.68(log10(ε˙ε0))0.1(fcof30)1.68

When the strain rate $\dot{\varepsilon }$ is equal to *ε*_0_ (10^−5^/s), Equation (11) is equal to Equation (10), and the hardening modulus model ${\bar{E}}_{2}$ degenerates into that for the FRP-confined pre-damaged concrete under quasi-static loading. The performance of Equation (11) is shown in Figure 12b, in which *AV* = 1.06 and *IAE* = 0.18. Therefore, Equation (11) can be used to calculate the hardening modulus value ${\bar{E}}_{2}$.

.

### 5.2. The Performance of the Proposed Model

**Figure 13:**
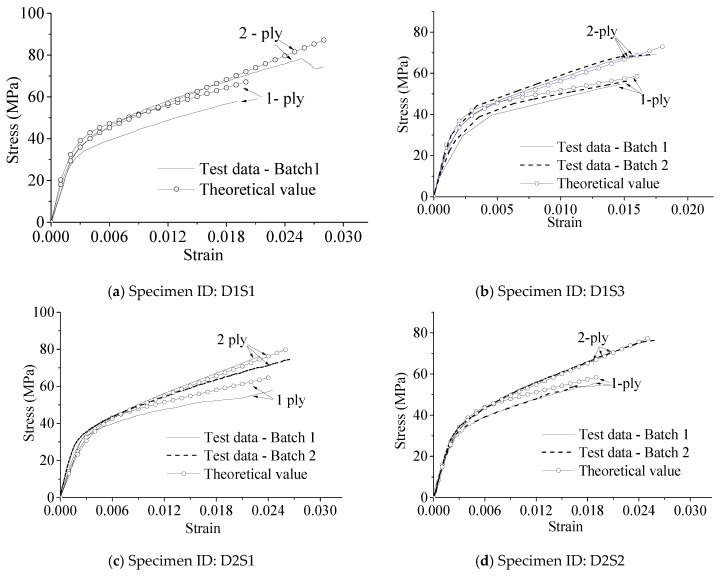

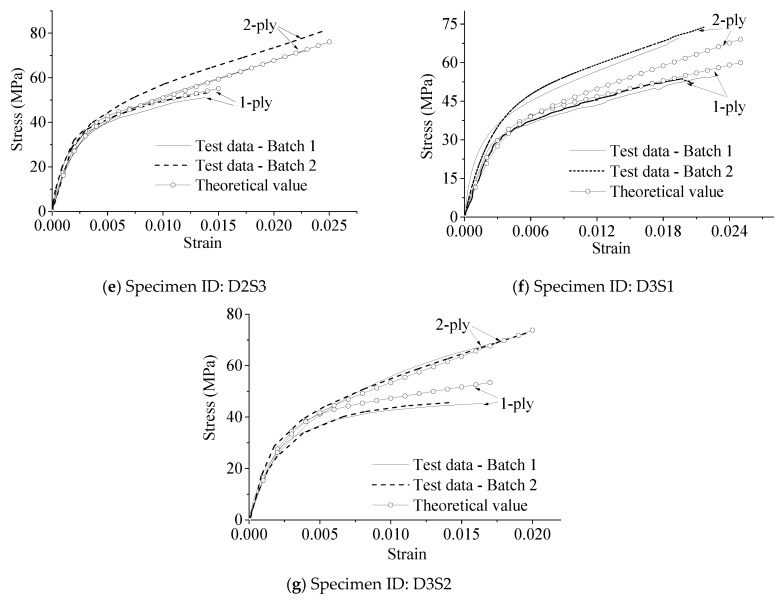
Performance of the proposed model.

The proposed stress–strain model consists of Equations (5), (7), (9), and (11) and the $\bar{n}$ value. Theoretical stress–strain curves can be drawn by using the proposed model. Figure 13 shows a performance comparison between the test data and the proposed model. In general, the agreement is satisfactory. Thus, the stress–strain relationship for the FRP-confined pre-damaged concrete columns can be described by the proposed stress–strain model. 

## 6. Conclusions

This work is an experimental and analytical investigation of FRP-confined pre-damaged concrete columns tested under varying strain rates. Based on the results, the following conclusions can be drawn from the study:

1. The experimental stress–strain curves of the FRP-confined damaged concrete under varying strain rates have a similar shape. Strain rates have significant effects on the initial Young’s modulus and hardening stiffness of stress–strain curves. With increasing strain rate, the initial elastic stiffness and hardening stiffness corresponding to the stress–strain curve also increase.
2. Strain rate has a significant effect on the ultimate strain; its value decreases with increases in the strain rate. The degree of concrete damage also has an effect on the ultimate strain and stress. Ultimate strain and stress values decrease with increases in the damage degree *δ*.
3. A stress–strain model of FRP-confined pre-damaged concrete under different strain rates is developed. After comparison with the test data, it is found that the proposed model can predict the characteristic points and the whole stress–strain curves.

## Figures and Tables

**Table 1:** Details of test specimens.

Specimen ID	*f_co_* (MPa)	*f_un_* (MPa)	*f_cd_* (MPa)	*δ*	*f_cu_* (MPa)	*ε_cu_*	*E*_1_ (GPa)	*n*	*f_o_* (MPa)	*E*_2_ (GPa)
* D0L1S1	27	0	27	0	-	-	22.3	0.7	21	2.8
* D0L2S1	27	0	27	0	-	-	19.8	0.7	21	4.1
D1L1S1-1	25	25	23.4	0.05	58	0.019	16.6	0.6	32.2	1.4
^#^ D1L1S1-2	27	27	25.4	0.06	57	-	-	-	-	-
D1L1S3-1	27	27	25.2	0.06	55	0.015	35.3	0.6	33.8	2.5
D1L1S3-2	26	26	24.4	0.06	56	0.015	23.1	0.7	34	1.5
D1L2S1-1	28	28	26	0.07	69	0.026	14.7	0.6	39.5	1.5
^#^ D1L2S1-2	28	28	26.4	0.06	78	-	-	-	-	-
D1L2S3-1	24	24	21.9	0.08	69	0.018	27.6	0.8	37.9	1.9
D1L2S3-2	27	27	25.1	0.06	70	0.017	30.6	0.8	40.6	1.8
^#^ D2L1S1-1	26	24	20.3	0.23	57	-	-	-	-	-
D2L1S1-2	27	25	21	0.23	58	0.025	17.2	0.7	30.7	1.3
D2L1S2-1	26	24	19.8	0.24	55	0.019	18.6	0.8	32.7	1.3
D2L1S2-2	29	26	21	0.27	54	0.016	16	0.6	31.8	1.4
D2L1S3-1	28	24	19.3	0.3	51	0.014	23.3	0.6	32	1.5
D2L1S3-2	29	25	19.9	0.3	53	0.014	24.3	0.7	32	1.8
D2L2S1-1	27	25	20.4	0.24	78	0.023	18.2	0.8	32.7	1.9
D2L2S1-2	26	23	18.9	0.27	75	0.026	24.1	0.8	35.4	1.5
D2L2S2-1	27	25	20.3	0.26	66	0.019	17.2	0.8	33.1	1.9
D2L2S2-2	26	23	18.5	0.24	77	0.026	15.1	0.6	34.9	1.7
D2L2S3-1	29	27	22.1	0.24	80	0.024	21.3	0.8	39.1	1.7
D2L2S3-2	30	27	21.2	0.3	70	0.023	19.7	0.8	34.3	1.7
D3L1S1-1	25	22	18.2	0.26	55	0.023	13.3	0.6	30	1.1
D3L1S1-2	29	21	10	0.65	54	0.020	12.9	0.6	29.9	1.2
D3L1S3-1	22	17	11.6	0.46	44	0.016	17.3	0.7	32	1.3
D3L1S3-2	28	20	9.2	0.67	45	0.014	17	0.6	30.4	1.4
D3L2S1-1	25	20	13.1	0.47	70	0.019	19.2	0.8	34.5	1.8
D3L2S1-2	27	21	13.8	0.49	74	0.022	15.7	0.8	35	1.9
D3L2S3-1	29	23	15.3	0.47	71	0.020	20.6	0.7	34.7	2
D3L2S3-2	25	19	11.3	0.55	69	0.018	16.2	0.8	35.1	2

* The values are determined by regressing the theoretical stress–strain model curve [29]. ^#^ Due to operational errors that occurred, only the load value is recorded.

**Table 2:** Material properties of carbon fiber-reinforced polymer (CFRP).

Strain Rate (strain/s)	Tensile Strength (MPa)		Rupture Strain		Elastic Modulus (GPa)	
	Test	Mean	Test	Mean	Test	Mean
3.3 × 10^−3^	4594	4566	0.0147	0.015	314	304
3.3 × 10^−4^	4662		0.0157		299	
3.3 × 10^−5^	4444		0.0147		299	
